# Expression of CCL2/CCR2 signaling proteins in breast carcinoma cells is associated with invasive progression

**DOI:** 10.1038/s41598-021-88229-0

**Published:** 2021-04-22

**Authors:** Wei Bin Fang, Diana Sofia Acevedo, Curtis Smart, Brandon Zinda, Nadia Alissa, Kyle Warren, Garth Fraga, Li-Ching Huang, Yu Shyr, Wei Li, Lu Xie, Vincent Staggs, Yan Hong, Fariba Behbod, Nikki Cheng

**Affiliations:** 1 Department of Pathology and Laboratory Medicine, University of Kansas Medical Center, Kansas City, KS 66160 USA; 2 Vanderbilt University Medical Center, Center for Quantitative Sciences, Nashville, TN 37232 USA; 3 Shanghai Center for Bioinformation Technology, Shanghai Academy of Science and Technology, Shanghai, 201203 People’s Republic of China; 4 Health Services and Outcomes Research, Children’s Mercy Hospital and Clinics, University of Missouri Kansas City School of Medicine, Kansas City, KS 64108 USA

**Keywords:** Breast cancer, Cancer models, Oncogenes, Tumour biomarkers

## Abstract

Ductal carcinoma in situ (DCIS) is the most common type of pre-invasive breast cancer diagnosed in women. Because the majority of DCIS cases are unlikely to progress to invasive breast cancer, many women are over-treated for DCIS. By understanding the molecular basis of early stage breast cancer progression, we may identify better prognostic factors and design treatments tailored specifically to the predicted outcome of DCIS. Chemokines are small soluble molecules with complex roles in inflammation and cancer progression. Previously, we demonstrated that CCL2/CCR2 chemokine signaling in breast cancer cell lines regulated growth and invasion through p42/44MAPK and SMAD3 dependent mechanisms. Here, we sought to determine the clinical and functional relevance of CCL2/CCR2 signaling proteins to DCIS progression. Through immunostaining analysis of DCIS and IDC tissues, we show that expression of CCL2, CCR2, phospho-SMAD3 and phospho-p42/44MAPK correlate with IDC. Using PDX models and an immortalized hDCIS.01 breast epithelial cell line, we show that breast epithelial cells with high CCR2 and high CCL2 levels form invasive breast lesions that express phospho-SMAD3 and phospho-p42/44MAPK. These studies demonstrate that increased CCL2/CCR2 signaling in breast tissues is associated with DCIS progression, and could be a signature to predict the likelihood of DCIS progression to IDC.

## Introduction

Ductal carcinoma in situ (DCIS) is the most common type of pre-invasive breast cancer, with approximately 60,000 cases diagnosed in women in the US every year. DCIS lesions are the precursor of invasive ductal carcinoma (IDC), and are characterized by the growth of neoplastic cells within the lumen of breast ducts. Currently, DCIS patients receive a standard treatment regimen involving a combination of radiation therapy and surgery, and in some cases, adjuvant anti-hormonal therapy^1,2^. Since most DCIS cases are unlikely to progress to invasive breast cancer, many women are over-treated for DCIS and experience a significant reduction in quality of life. Conversely, up to 20% of patients treated for DCIS experience disease recurrence, often accompanied by invasive breast cancer, indicating that current treatment strategies are not sufficient for a subset of DCIS cases^1,2^. There are few approaches to evaluate the prognosis of DCIS. Small or low-grade DCIS lesions, which are considered low risk, may still become invasive^3–5^. Expression of proliferation and hormone related genes are associated with DCIS recurrence, but not with development of invasive breast cancer^6,7^. Relative to invasive breast cancer, biomarkers in DCIS have been understudied. By understanding the molecular basis of early stage breast cancer progression, we may identify better prognostic factors and design treatments more tailored to the outcome of DCIS.

Chemokines are soluble proteins (8 kda) with complex roles in inflammation and cancer progression. They form molecular gradients to mediate homing and activity of immune cells during inflammation. Chemokines signal to G protein coupled receptors to promote release of inflammatory mediators, cell proliferation, adhesion and migration. Chemokines and their receptors are subdivided into C–C, CXC, CXC3C or XC classes, depending on the composition of a conserved amino acid motif involved in ligand/receptor binding^8,9^. The C–C class of chemokines are important for homing and activity of T cells and macrophages. C–C Ligand 2 (CCL2) and its primary receptor CCR2 are particularly important for regulating macrophage recruitment during wound healing and infection^10^. CCL2 is overexpressed in various cancers including glioma, prostate and breast cancers^11–14^. In invasive breast cancer, CCL2 expression in the stroma correlates with disease recurrence^13,14^. Knockdown or antibody neutralization of CCL2 in breast xenograft models inhibits infiltration of CCR2+ macrophages and reduces tumor growth and metastasis^13,15,16^. These studies indicate an important role for CCL2 expression in late stage cancers.

Recent studies have implicated a role for CCL2 and CCR2 in progression of early stage breast cancers. For one, CCR2 expression in breast cancer cell lines correlates with their invasive potential^17^. Additionally, in vitro studies have demonstrated that CCL2/CCR2 mediated p42/44MAPK and SMAD3 pathways in breast cancer cells are important in growth, survival, migration and invasion^17,18^. In a mouse mammary intraductal injection (MIND) model that mimics DCIS formation in patients, the functional role of CCR2 has been explored in two human breast cancer cell lines: SUM225, a lowly invasive cell line, and the highly invasive DCIS.com. Stable overexpression of CCR2 in SUM225 breast cancer cells enhances progression of DCIS lesions to invasion. Conversely, CCR2 shRNA knockdown or ablation by CRISPR knockout in DCIS.com breast cancer cells inhibits the number of invasive lesions, limiting DCIS progression^18^. These studies demonstrate that CCR2 expression is important in DCIS progression and identify CCL2/CCR2 as a regulator of SMAD3 and p42/44MAPK signaling in breast cancer cells. However, these studies have mainly involved transformed cell lines and have not addressed the physiologic or clinical relevance of CCL2/CCR2 signaling to DCIS progression. Addressing these questions would provide an important justification to further develop the CCL2/CCR2 pathway as a predictive signature and in approaches to prevent invasive breast cancer.

Here, we determined the physiologic and clinical relevance of CCL2/CCR2 signaling proteins to DCIS progression using a combination of patient tissues, PDX models and immortalized breast epithelial cells. Through immunostaining analysis of DCIS, IDC and normal tissues, we showed that expression of CCL2, CCR2, phospho-SMAD3 and phospho-p42/44MAPK correlated with IDC. Using PDX models and an immortalized hDCIS.01 breast epithelial cell line, we demonstrated that breast epithelial cells with high CCR2 and high CCL2 levels form invasive breast lesions that express phospho-SMAD3 and phospho-p42/44MAPK. Overall, these studies demonstrate that increased CCL2/CCR2 signaling in breast cancer cells is a physiologically and potentially clinically relevant signature in DCIS progression.

## Material and methods

### Ethical approval

All experiments involving human tissues were approved and performed under guidelines and regulations by the ethics review board at the University of Kansas Medical Center (KUMC). The tissues collected for tissue microarrays were categorized as Exempt. Tissue samples were de-identified by the Biospecimen Core Repository Facility (BCRF) prior to distribution. Written informed consent for tissue collection for immunostaining studies was obtained by the BCRF, which has IRB approval. For specimens used for in vivo injections, written informed consent to participate in this research was obtained under an IRB approved protocol. Medical records were used in compliance with KUMC regulations, aligned with the World Medical Association Declaration of Helsinki.

Animal experiments were performed at KUMC under an approved institutional animal care and use committee protocol. Animals were cared for in accordance with the Association for Assessment and Accreditation of Laboratory Animal Care. The study was carried out in compliance with the ARRIVE guidelines.

### Cell isolation/culture

Research samples were collected from patients undergoing image guided core needle biopsy or surgical excision, after collection of diagnostic specimens. Primary cells were processed for mammary intraductal injection as described previously^19^. h.DCIS.01 breast epithelial cells were cultured as described^20^. To enrich for CCR2+ cells, hDCIS.01 cells were detached from plates using Accutase (Millipore, cat no.SCR005). 5 million cells were incubated with 500 μl Protein A beads (Thermo Scientific, cat no. 100552311) conjugated to CCR2 antibody (R&D Systems, cat no. MAB150) at 4 °C for 30 min. Cells bound to beads were washed with PBS and cultured in 10 cm dishes. Clones were isolated using cloning discs and amplified. SUM225 cells stably overexpressing CCR2 (CCR2-H) were generated and cultured as previously described^18^. Cells were maintained at most 3 months at a time. Cells were tested for mycoplasma by MycoAlert (Lonza cat no. LT07-118).

### Mouse Mammary intra-ductal (MIND) injection

Non-obese Diabetic Severe Combined Immunodeficient interleukin receptor-γ2 null mice (NOD-SCID IL-2rγ−/−), 8–10 weeks old were purchased from Jackson Laboratories. MIND injections were performed using procedures described previously^21^. For CCL2 delivery, dosage was based on previous studies^22^, and were optimized for the mammary gland through pilot studies. 90 day release pellets containing 500 ng recombinant human CCL2 were custom made from Innovative Research of America. An incision was made to expose the #4–5 and #9–10 mammary glands. A placebo control or CCL2 pellet was implanted into each of the inguinal mammary fat pads after injection of cells. The skin flaps were closed using gut absorbable suture. Mice were monitored twice a week and euthanized 22 weeks post-injection. Depending on available cells, 4 mice were injected each with case nos. IC-041717-1 and IC-031317. Two mice were injected with case no. IC-022316.

### Tissue microarrays (TMA)

Biomarker studies were conducted using REMARK criteria^23^. TMAs containing DCIS core sections (n = 19 cases) were obtained from US Biomax (cat no.BB08015). TMAs were generated by the BCRF at the University of Kansas Medical Center (KUMC). These TMAs included: DCIS (n = 53 cases) with matching normal adjacent breast tissues (n = 25), independent IDC cases (n = 74 cases) with matching normal adjacent breast tissues (n = 61 cases). Table 1 summarizes de-identified patient information on tissues obtained from the BCRF. TMAs were arrayed in duplicate, with core sections of 1.5 mm in diameter, 5 microns thickness. DCIS was graded according to Van Nuys Prognostic Index (VNPI). Cases of IDC were evaluated according to Scarff-Bloom and Richardson (SBR) grading system. Samples were collected prior to treatment between 2007 and 2012, with 3–5 years follow-up. Seven deaths were reported in the IDC cohort, a number insufficient for statistical analysis. Based on St Gallen’s criteria^24^, luminal A breast cancers were defined as ER+ and/or PR+, HER2−, < 20% Ki67. Luminal B breast cancers were defined as ER and/or PR+, HER2+ or HER2−, ≥ 20% Ki67. HER2+ were characterized by ER−, PR− and HER2+. Triple negative breast cancers were identified as ER−, PR− and HER2−.

**Table 1:** Clinical features for DCIS and IDC patient samples from the Biospecimen Core Repository Facility.

Factor	DCIS (n = 53)	IDC (n = 74)
**Race**		
Black	8%	5%
White	62%	91%
Asian/other	30%	4%
Median age	52	55
ER	4.6^a^ 65.3^b^ 98^c^	90^a^ 99^b^ 100^c^
PR	0^a^ 27.5^b^ 86.5^c^	1.5^a^ 85^b^ 100^c^
**HER2**		
0	27%	51%
1	42%	44%
2	12%	0%
3	19%	5%
Median Ki67	6	5

Percent of total are shown with actual number of cases in parentheses.

^a^25% percentile, ^b^median, ^c^75th percentile are shown for Estrogen Receptor (ER) and Progesterone Receptor (PR).

### Flow cytometry

Cells were immunostained with CCR2 antibodies as previously described^17^. Briefly, cells were incubated with anti-CCR2-PE (Biolegend, cat no. 357205) for 1 h, and analyzed using a LSRII Flow cytometer. Expression was normalized to unstained controls.

### Immunohistochemistry

Tissues were processed into paraffin and immunostained as previously described^25^. Slides were incubated overnight with antibodies to: CCL2 (Santa Cruz Biotechnology cat no. 1304) for DAB staining, CCL2 (Biolegend cat no. 502602) for immunofluorescence (IF) staining, CCR2 (Santa Cruz Biotechnology cat no. sc7395), phospho-SMAD3 s423/s425 (Cell Signaling Technology cat. no. 9520), phospho-p42/44MAPK Thr202/Thr204 (Cell Signaling Technology cat no. 4376), or PCNA (Biolegend cat no. 307902).

For DAB staining, CCL2 expression was detected by anti-goat biotinylated antibodies (Vector Laboratories cat no.BA5000). CCR2, phospho-p42/44MAPK and phospho-SMAD3 proteins were detected by anti-rabbit-biotinylated antibodies (Vector Laboratories, cat no. BA1000). Slides were incubated with streptavidin peroxidase (Vector Laboratories cat no. PK-4000), visualized with 3,3′-Diaminobenzidine substrate (DAB) (Vector Laboratories cat no. SK-4100), counterstained with Harris’s hematoxylin and mounted with Cytoseal (Richard-Allen Scientific cat no. 8310-16).

For IF, slides were incubated with anti-rabbit-IgG-Dylight-488 (ThermoFisher cat no.35552) to detect α-sma, CCR2, phospho-SMAD3 or phospho-p42/44MAPK, anti-mouse IgG-AlexaFluor-647 (ThermoFisher cat no. A28181) to detect CK5/CK19, anti-mouse IgG-Dylight-488 (ThermoFisher cat no. 35502) to detect PCNA or anti-mouse-biotinylated followed by streptavidin-AlexaFluor-568 (Invitrogen cat no. S11226) to detect CCL2. Sections were counterstained with 4′,6-diamidino-2-phenylindole (DAPI) and mounted with 1:1 PBS/glycerol. 5 images were captured using an EVOS FL Auto Imaging System (Invitrogen).

Specificity of anti-CCL2 was demonstrated previously^25^. Specificities of CCR2, phospho-SMAD3 and phospho-p42/44MAPK antibodies were demonstrated through competition assays with peptides added at a tenfold excess to primary antibodies (Supplementary Fig. S1): CCR2 peptides (Novus Biologicals cat no. NBP1-48337PE), Phospho-P42/44MAPK peptides (Cell Signaling Technology cat no. 1150s), Phospho-SMAD3 peptides (Abcam cat no. ab135224). Species specificity was controlled for using MMTV-PyVmT mouse mammary tumor samples generated previously^26^.

### Image quantitation by software

Biomarker expression was quantified using methods described previously^14^. Briefly, images were imported into Adobe Photoshop. DAB or fluorochrome staining was selected, copied and saved as a separate file. The images were opened in Image J, and subject to particle analysis. Positive immunostaining was expressed as particle area values of arbitrary unit and normalized to total hematoxylin or DAPI staining.

### Manual scoring

DCIS and IDC samples were immunostained for CCL2 and phospho-p42/44MAPK and provided to a clinical pathologist for blinded numerical scoring. CCL2 and phospho-p42/44MAPK levels were scored as negative/weak (1), moderate (2) or strong (3). Two–three serial sections from MIND model samples were scored for invasion in a blinded fashion. Non-invasive (1), indicated no to low invasion, characterized by intact myoepithelium and confinement of epithelial cells within the duct. Invasive (2), indicated 20% or more disappearance of the myoepithelium and/or 1 or more epithelial cells contacting the periductal stroma.

### 3D cell culture

3D cultures were established and analyzed as previously described^27^. Cells were treated with/without 100 ng/ml human recombinant CCL2 (Peprotech cat no. 300-04) or 20 nM INCB3284 (Cayman Chemical cat no. 11963) in growth media containing 2.5% Matrigel. Images were captured at day 4 for hDCIS.01 cells and day 8 for SUM225 cells at 10× magnification, 4 fields/well using an EVOS FL Auto Imaging system. Spheroid size was quantified using Image J software.

### Immunoblot analysis

Immunoblot analysis was performed as previously described^27^. Cells were treated with/without 100 ng/ml CCL2 and/or 20 nM INCB3284 for up to 30 min. Nitrocellulose membranes were probed with antibodies to: SMAD3 (Cell Signaling Technology, cat no. 9523), phospho-SMAD3 (Ser-423/425, cat no. 9520, Cell Signaling Technology), phospho-p42/44MAPK (Thr202/Tyr204, Cell Signaling Technology, cat no. 4376), p42/44MAPK (Cell Signaling Technology, cat no. 9102), or β-ACTIN (Sigma, cat no. A5441). Chemiluminescence was captured using the UVP Imaging System.

### Statistical analysis

Sample sizes were determined using PS Power and Sample Size Ver3.0. Minimum sample size was determined to be 64 cases of DCIS and IDC to yield meaningful data with 80% power with alpha = 0.05. Patient sample populations did not fit a normal distribution and were uneven due to two factors. Information on prognostic factors was not available for US Biomax samples. Some sections did not adhere to slides during staining, affecting sample size. Therefore, protein expression values and their relationships to clinical data were analyzed using non-parametric methods.

Statistical analysis was performed using GraphPad Prism and SAS software. For data exhibiting non-normal distribution, Mann–Whitney Wilcoxon test was used to compare two independent sample populations. Wilcoxon Signed Rank Test was used to compare two dependent sample populations. For more than two groups, Kruskal Wallis Test with Dunn’s post-hoc comparison was used. Associations with continuous variables were analyzed using Spearman correlation test. For data exhibiting normal distributions, Two Tailed t-test or One-Way ANOVA with Bonferroni post-hoc comparison was used. Associations with invasion were analyzed using Chi-Square test. Incidence of tumor formation was analyzed using Kaplan Meier curve and Log-Rank (Mantel-Cox) test. Statistical significance was determined by p < 0.05. n.s = not significant.

## Results

### Increased expression of CCL2/CCR2 signaling proteins in DCIS and IDC

To determine the clinical relevance of CCL2/CCR2 signaling protein expression in breast cancer, we performed immunohistochemistry staining on patient samples of DCIS, IDC and normal adjacent tissues for detection of CCL2, CCR2, phospho-SMAD3 and phospho-42/44MAPK proteins. While DAB staining is typically scored manually, we chose to quantify staining using Image J software^14^ because continual values were produced, which enabled more thorough statistical evaluation over manual scoring. Image J values corresponded closely to manual scoring of staining intensity (Supplementary Fig. S2A,B), indicating this software approach was just as reliable as manual scoring.

**Figure 1:**
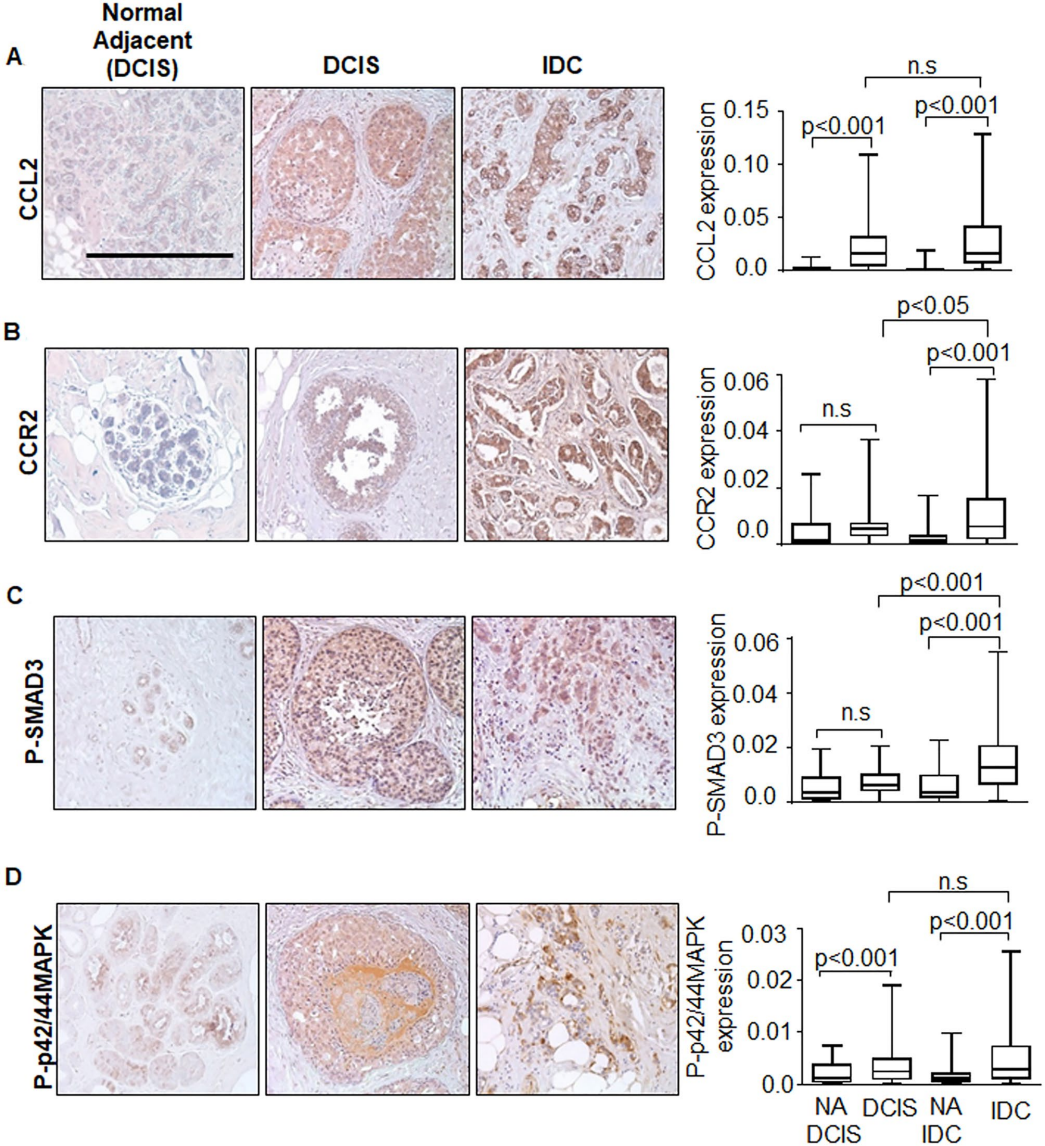
Expression of CCL2/CCR2 related signaling proteins are elevated in DCIS and further upregulated in IDC patient tissues. TMAs containing DCIS (n = 72), IDC (n = 74) or normal adjacent breast tissues to DCIS (NA DCIS) (n = 25) or to IDC (NA IDC) (n = 61) were immunostained with antibodies to: (**A**) CCL2, (**B**) CCR2, (**C**) phospho-SMAD3 or (**D**) phospho-p42/44MAPK. Scale bar = 200 microns. Immunostaining was quantified by Image J. Whisker box plots are shown. Whiskers indicate min and max values. Box indicates upper and lower quartile range. Line indicates median. Statistical analysis for DCIS vs. IDC was performed using Mann–Whitney–Wilcoxon Test. Statistical analyses for NA DCIS vs. DCIS and NA IDC vs. IDC were performed using Wilcoxon Signed Rank Test. Statistical significance analysis was determined by p < 0.05. *ns* not significant. Scale bar = 200 microns.

Expression levels of CCL2, CCR2, phospho-SMAD3 and phospho-p42/44MAPK were analyzed in DCIS, IDC and matching normal adjacent tissues. Increased CCL2 and phospho-p42/44MAPK expression were detected in DCIS compared to normal tissues. CCL2, CCR2, phospho-SMAD3 and phospho-p42/44MAPK expression were increased in IDC tissues compared to normal breast tissues. Higher levels of CCR2 and phospho-SMAD3 expression were observed in IDC compared to DCIS tissues (Fig. 1A–D). Overall, some CCL2/CCR2 signaling proteins were elevated in DCIS, and an even greater number of CCL2/CCR2 proteins were overexpressed in IDC.

Next, we evaluated associations of CCL2, CCR2, phospho-SMAD3 and phospho-p42/44MAPK with commonly used prognostic factors. No associations were identified between CCL2/CCR2 signaling proteins and age, Ki67 or histologic grade (Supplementary Table S1, Supplementary Fig. S3A–D)**.** Expression of CCL2 inversely correlated with nuclear grade in IDC tissues (Supplementary Fig. S4A–D). In summary, there are few associations of CCL2/CCR2 signaling proteins to known prognostic factors.

We examined associations of CCL2, CCR2, phospho-SMAD3 and phospho-p42/44MAPK with molecular subtype including: luminal A and B, HER2+ and triple negative breast cancers, which were identified using clinical guidelines on ER, PR, HER2 and Ki67/PCNA expression^28–30^. The only association detected was a significant increase in phospho-SMAD3 expression in luminal A breast cancers compared to HER2+ breast cancers (Supplementary Fig. S5A–D). Overall, there was little association between CCL2/CCR2 signaling proteins and breast cancer subtype.

### Increased CCL2/CCR2 signaling in breast epithelial cells enhances formation of invasive lesions in PDX models

One limitation to analyzing biomarker expression in DCIS and IDC tissues was that these tissues came from different patients, preventing us from drawing predictive conclusions. While collecting cohorts of pure DCIS tissues with follow-up information on recurrence and survival is valuable, this process is labor intensive and takes many years. To follow the natural progression of DCIS, we used PDX mouse models. In previous studies, cells from patient derived DCIS cases formed breast lesions in the MIND model, which retained their original expression of cytokeratin, ER, PR and HER2^19^. Therefore, the MIND model represents a physiologically relevant approach to follow the progression of lesions expressing varying levels of CCR2.

To determine the functional relevance of CCL2 levels and CCR2 expression to DCIS progression, patient derived breast epithelial cells were isolated from DCIS tissues (Table 2) and injected via MIND model into NOD-SCID mice for up to 22 weeks. Mammary tissues were harvested and analyzed for changes in CCL2/CCR2 signaling and DCIS progression by IF. Human breast lesions were identified by expression of human CK5/CK19. The myoepithelium surrounding the breast duct was identified through α-sma expression. Invasive lesions were characterized by loss of α-sma+ myoepithelium and presence of CK5/19+ cells in the peri-ductal stroma. Cell proliferation was assessed by PCNA staining of CK5/19+ cells. Expression of CCL2/CCR2 signaling proteins were examined in CK5+/19+ lesions.

**Table 2:** Profile of de-identified patient samples used in mammary intraductal injection studies.

Patient sample	Diagnosis	Biomarker		
		ER	PR	HER2
IC-041717-1	DCIS low grade	+ 100%	+ 40%	NA
IC-031317	DCIS high grade, comedo/solid	+ 5%	−	NA
IC-022316	DCIS intermediate grade, micropapillary/sold	+ 99%	+ 68%	+

Patient diagnosis, ER, PR, HER2 data were provided in pathology reports. IC-041717-1 and IC-031317 were obtained by needle core biopsy. IC-022316 were obtained through surgical biopsy.

*NA* information not available.

**Figure 2:**
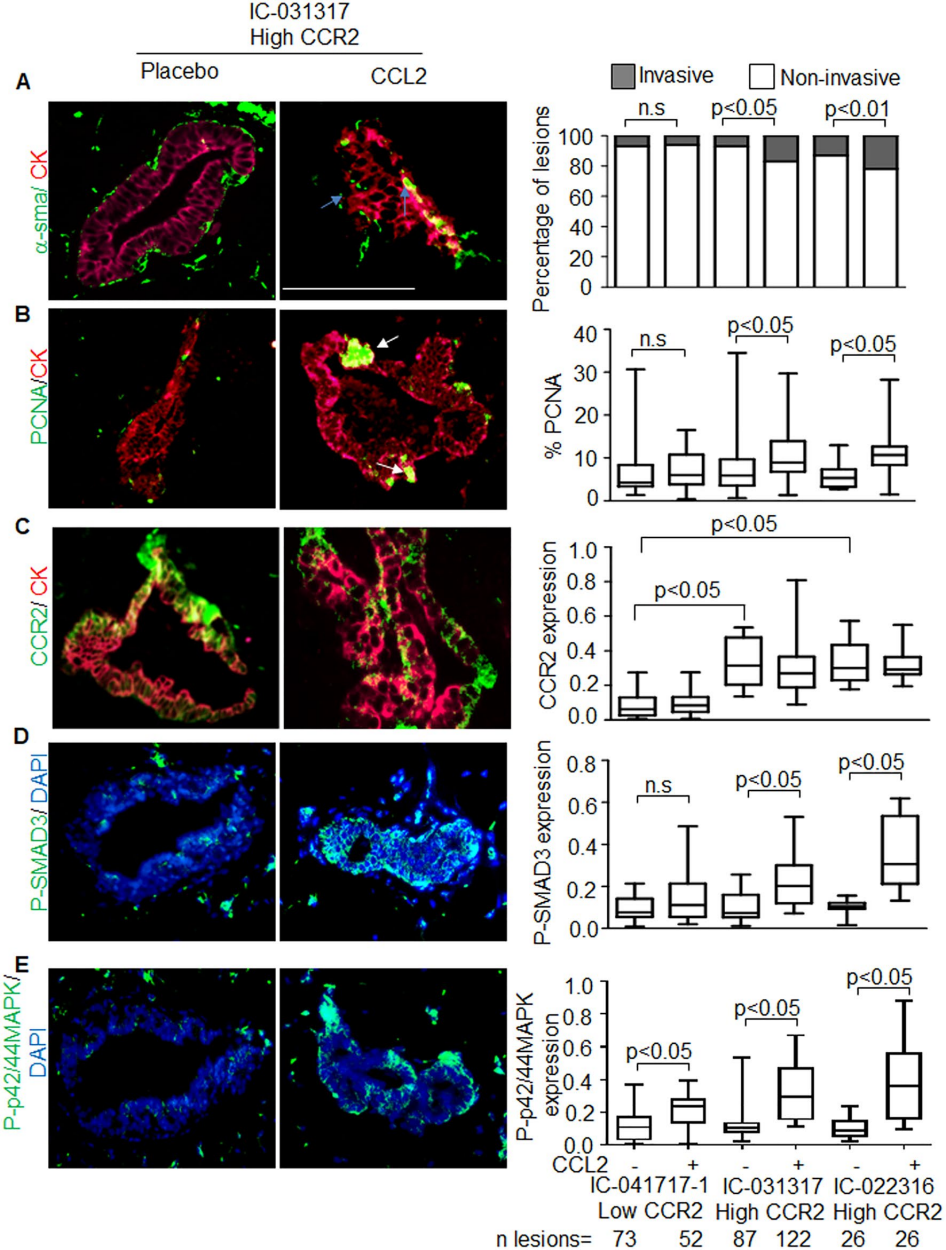
CCL2-mediated progression of patient derived breast xenografts is associated with increased CCR2, phospho-SMAD3 and phospho-p42/44MAPK expression. N = 4 mice (8 mammary fat pads total) were injected each with DCIS cells from patients IC-041717-1 and IC-031317. N = 2 mice (4 mammary fats total) were injected with DCIS cells from patient IC-022316. Mice were treated with placebo or recombinant CCL2 for up to 22 weeks. Primary breast xenografts were co-immunofluoresence stained for CK5/CK19 (red) with (**A**) α-sma, (**B**) PCNA, (**C**) CCR2, (**D**) phospho-SMAD3 (green) or E. phospho-p42/44MAPK (red). Sections were counter stained with DAPI. Representative images are shown for case no. IC-031317 with CK5/CK19 overlay with α-sma, PCNA or CCR2, and DAPI overlay with phospho-SMAD3 or phospho-p42/44MAPK expression. Blue arrows point to invasive carcinoma cells, characterized by absence of α-sma staining and presence of cancer cells in the peri-ductal stroma. White arrows point to positive PCNA staining. Scale bar = 200 microns. Sample size of mammary lesions per group are shown below the last graph. Invasiveness was scored for lesions co-stained for α-sma/CK5/19. Expression was quantified by Image J. Stacked bar graph is shown is shown for percentage of invasive and non-invasive lesions (**A**). Whisker box plots are shown for (**B**–**E**). Whiskers indicate min and max values. Box indicates upper and lower quartile range. Line indicates median. Statistical analysis was performed using Chi Square Test (**A**) or Two tailed T-test (**B**–**E**). Statistical significance analysis was determined by p < 0.05. *ns* not significant.

We examined the significance of CCR2 expression in breast lesions. Breast lesions showing higher CCR2 expression (IC-031317 and IC-022316) did not show significant differences in growth and invasion of breast lesions compared to those with relatively low CCR2 expression (IC-041717-1) (Fig. 2A–C, Supplementary Fig. S6A–C). Furthermore, CCR2 expression alone was not associated with changes in phospho-SMAD3 or phospho-p42/44MAPK expression in breast lesions. These data indicated that CCR2 expression alone or autocrine CCL2/CCR2 signaling in primary breast lesions did not affect growth and invasion.

Previous studies showed that CCL2 significantly stimulated CCR2 signaling in breast cancer cells^27^. Therefore, we hypothesized that CCL2 delivery would enhance DCIS progression to lesions with high CCR2 expression. While murine CCL2 may cross-react with human chemokine receptors^31^, the levels of CCL2 from murine mammary tissue^25^ may not be sufficient to induce CCR2 signaling in human breast epithelial cells. Therefore, we determined the effects of exogenous human CCL2 on formation of patient derived breast lesions by orthotopically implanting slow release pellets. CCL2 delivery was associated with a significant increase in the growth and invasion of IC-031317 and IC-022316 breast lesions, which had high CCR2 expression (Fig. 2A–C, Supplementary Fig. S6A–C). Growth and invasion of these lesions were associated with increased expression of phospho-SMAD3 and phospho-p42/44MAPK expression (Fig. 2A–E, Supplementary Fig. S6A–E). CCL2 treatment of IC-041717-1 lesions did not affect growth, invasion or expression of phospho-SMAD3 and phospho-p42/44MAPK (Fig. 2A–E, Supplementary Fig. S6A–E). CCL2 levels were elevated in mammary tissues implanted with CCL2 pellets over placebo treatment (Supplementary Fig. S7). Overall, DCIS cases IC-031317 and IC-022316, which showed higher CCR2 expression, formed more invasive breast lesions with CCL2 treatment, compared to IC-041717-1, which showed low CCR2 expression.

### CCR2+ hDCIS.01 cells form more invasive breast lesions in vivo

**Figure 3:**
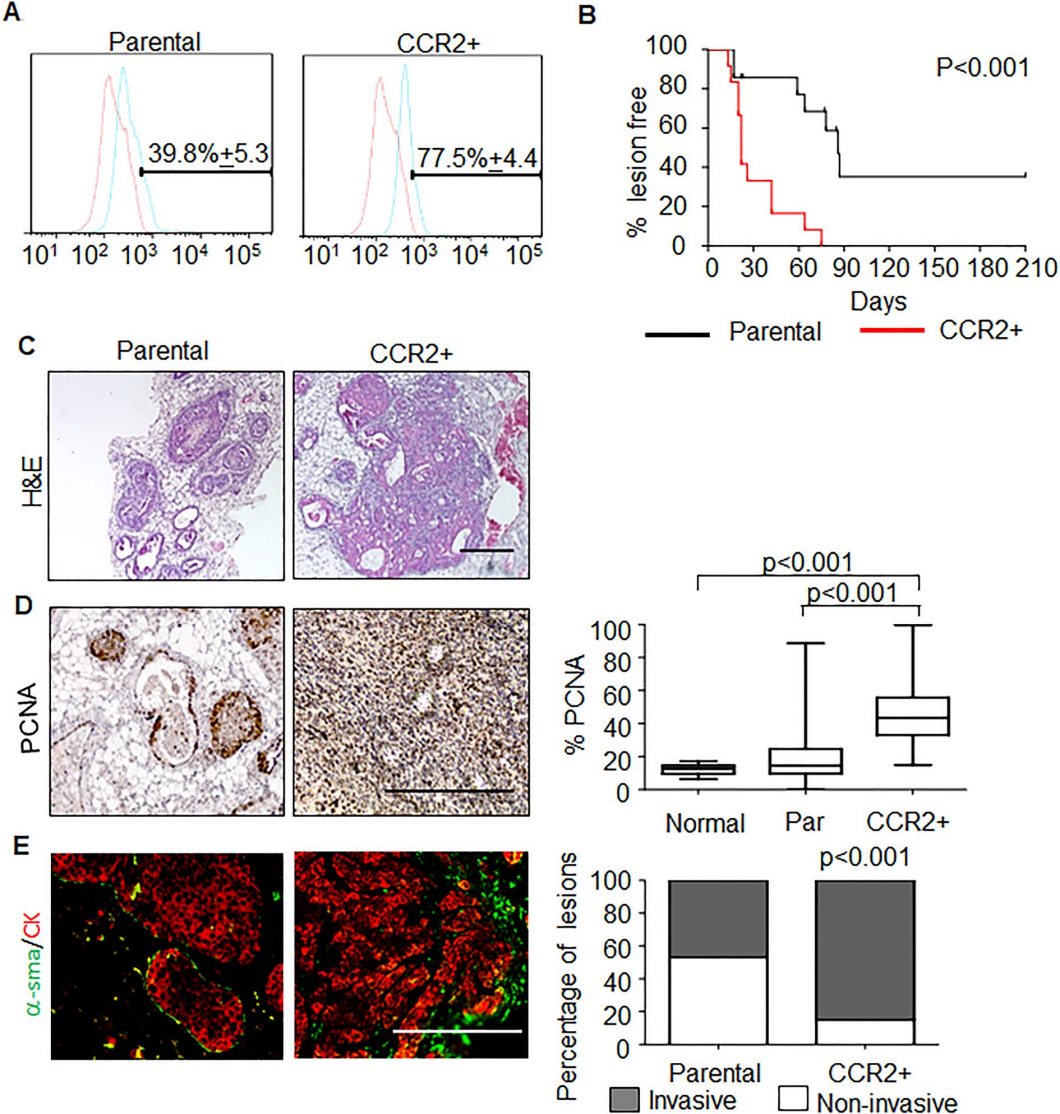
CCR2+ hDCIS.01 breast cancer cells show increased formation of invasive mammary lesions. (**A**) hDCIS.01 cells were magnetically sorted for CCR2 expression (CCR2+), and analyzed by flow cytometry for CCR2 expression 4 weeks post-enrichment. (**B**) Parental or CCR2+ cells were injected into the mammary ducts of NOD-SCID mice. Mice were palpated for tumor formation twice weekly. Tumor formation was plotted as a function of percent tumor free over time. Parental n = 14, CCR2+ n = 12. (**C**) H&E stain of mammary lesions from matching CCR2+ lesions and parental control harvested 75 days post-injection. (**D**) Cell proliferation was analyzed by PCNA immunostaining, normalized to hematoxylin staining. Whisker box plots are shown. Whiskers indicate min and max values. Box indicates upper and lower quartile range. Line indicates median. (**E**) Invasiveness was scored for lesions co-stained for α-sma/CK5/19. Statistical analysis was performed using Log Rank (Mantel-Cox) Test (**B**), One Way ANOVA with Bonferroni post-hoc comparison (**D**) or Chi Square test (**E**). Statistical significance was determined by p < 0.05. Scale bar = 200 microns.

Because CCR2 is expressed in a subset of breast epithelial cells, we sought to further determine the functional relevance of this subset to DCIS progression using hDCIS.01 cells, an immortalized cell line derived from primary hyperplastic breast epithelial cells^20^. CCR2+ cells were magnetically sorted from the h.DCIS.01 cell line and expanded in culture for 4 weeks, before analysis by flow cytometry. Compared to parental cells, CCR2 expression remained higher in CCR2+ cells, indicating that CCR2+ cells were stable (Fig. 3A).

To examine the functional relevance of CCR2+ subsets, we injected these cells into the mammary ducts of NOD-SCID and palpated for formation of breast lesions over time. Cells enriched for CCR2 expression formed breast lesions at earlier times, compared to control parental cells (Fig. 3B). Histological analysis showed that CCR2+ lesions were larger in size and had a more reactive stroma compared to parental lesions (Fig. 3C). Lesions enriched for CCR2+ displayed higher levels of PCNA compared to parental lesions and normal tissue (Fig. 3D). Additionally, tissues enriched for CCR2+ displayed a significantly higher percentage of invasive lesions compared to parental (Fig. 3E). These data indicated that the CCR2+ breast carcinoma cells were associated with increased proliferation and formation of invasive breast carcinomas.

**Figure 4:**
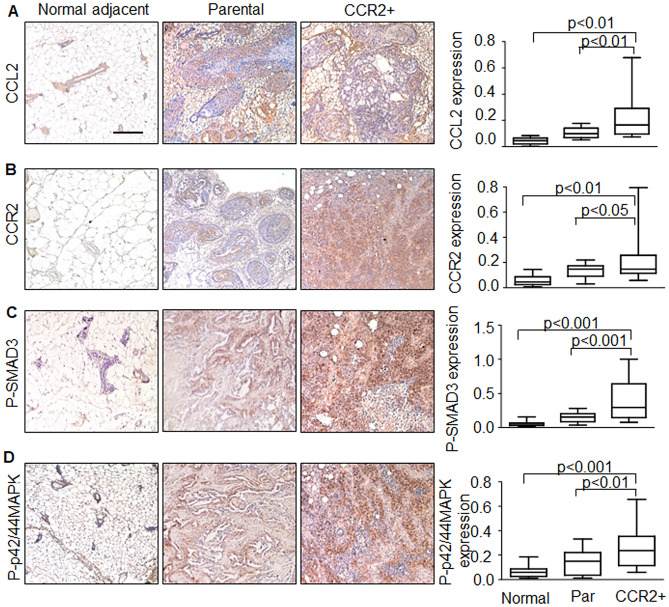
hDCIS.01 xenografts enriched for CCR2 expression show increased CCL2, phospho-SMAD3 and phospho-p42/44MAPK expression. hDCIS.01 xenografts were immunostained for expression of: (**A**) CCL2, (**B**) CCR2, (**C**) phospho-SMAD3 or (**D**) phospho-p42/44MAPK. Normal adjacent tissues were from parental lesions. Normal adjacent n = 12, parental n = 14, CCR2+ n = 12. Expression was quantified by Image J. Statistical analysis was performed using One Way ANOVA with Bonferroni post-hoc comparison. Whisker box plots are shown. Whiskers indicate min and max values. Box indicates upper and lower quartile range. Line indicates median. Statistical significance was determined by p < 0.05. Scale bar = 200 microns.

We then examined expression of CCL2/CCR2 signaling proteins in hDCIS.01 breast lesions. Parental lesions showed higher expression of CCL2, CCR2, phospho-SMAD3 and phospho-p42/44MAPK proteins compared to normal adjacent tissues. CCR2+ lesions displayed even higher expression of CCL2, phospho-SMAD3 and phospho-p42/44MAPK proteins compared to parental and normal adjacent tissues (Fig. 4A–D). Delivery of CCL2 did not further accelerate lesion formation of CCR2+ cells injected in the mammary ducts of mice (Supplementary Fig. S8). Overall, these data indicated that ductal carcinomas with high CCR2 expression were associated with increased invasiveness and increased expression of CCL2, phospho-SMAD3 and phospho-p42/44MAPK.

To determine the feasibility of targeting CCR2 on breast cancer cell growth, we used a 3D culture approach, useful for modeling drug efficacy^32,33^. We examined the effects of a CCR2 antagonist INCB3284, which inhibits binding to CCL2 (IC50 = 3.7 nM)^34^. Compared to parental hDCIS.01 cells, CCR2+ cells showed increased growth of spheroids, which was further enhanced with CCL2 treatment. Treatment with 20 nM INCB3284 in 3D cultures inhibited CCL2-mediated growth of CCR2+ hDCIS.01 spheroids by approximately 50% (Supplementary Fig. S9A). INCB3284 treatment also inhibited CCL2-mediated spheroid growth of CCR2-H SUM225 cells, supporting the effects of CCR2 inhibition (Supplementary Fig. S9B). INCB3284 treatment inhibited CCL2-induced phosphorylation of SMAD3 and p42/44MAPK and decreased the overall protein levels of p42/44MAPK in both cell lines (Supplementary Fig. S9C,D; full-length blots shown in Supplementary Figs. S10 and S11). Overall, these studies demonstrate that CCR2 antagonists impair CCL2/CCR2 mediated growth and signaling of breast cancer cells in vitro.

## Discussion

Previous studies have demonstrated that increased CCL2/CCR2 mediated activation of p42/44MAPK and SMAD3 signaling regulates growth, survival, migration and invasion of transformed breast cancer cell lines^17,18^. Through analysis of patient derived breast tissues and mouse models of DCIS, we showed that expression of CCL2, CCR2, phospho-SMAD3 and phospho-p42/44MAPK proteins were associated with invasive breast ductal carcinomas. Overall, these studies enhance our understanding of the underlying molecular basis of DCIS progression, and could lead to new approaches to predict DCIS progression, and therefore influence the course of treatment for DCIS patients.

In studies of patient tissues, DCIS and IDC were distinguished from normal breast by showing increased CCL2 and phospho-p42/44MAPK expression. IDC tissues were distinguished from DCIS by showing higher expression of CCR2 and phospho-SMAD3. These data indicated that expression of CCL2/CCR2 signaling proteins correlated with DCIS progression to IDC. In the MIND model, CCL2 treatment of patient derived breast lesions (IC-041717-1) enhanced phospho-p42/44MAPK expression but did not affect cell proliferation or invasion. These lesions still expressed CCR2 but at lower levels compared to IC-031317 and IC-022316 primary lesions. These data suggested that increased CCL2/CCR2 mediated p42/44MAPK signaling were not sufficient to promote DCIS progression, but required additional pathways to increase growth and invasion. Previous studies have shown that SMAD3 regulates mammary tumor growth, invasion and metastasis in mouse models^35,36^. Furthermore, SMAD3 cooperation with p42/44MAPK was found to be important in CCL2/CCR2 mediated breast cancer cell survival and motility^17^. Here, phospho-SMAD3 expression was associated with increased CCL2 and CCR2 levels, and increased growth and invasion of patient derived lesions (IC-031317, IC-022316) and hDCIS.01 breast lesions in mice. These data suggested that increased CCR2 expression in breast epithelial cells could amplify the effects of CCL2 signaling and activate additional downstream signaling pathways such as SMAD3 to enhance formation of invasive breast lesions.

PDX models allowed us to follow DCIS progression in a physiologically relevant manner. These studies were complemented by the hDCIS.01 MIND model, which enabled us to examine the contribution of endogenous CCR2 expression to DCIS progression. We noted some consistent trends between both models. In the hDCIS.01 and primary DCIS cases examined in the MIND models, CCR2 expression was associated with increased growth and invasion of breast lesions and increased phospho-p42/44MAPK and phospho-SMAD3 expression. Interestingly, CCL2 delivery to CCR2 highly expressing primary xenografts enhanced formation of invasive lesions. This was not true of CCL2 delivery to CCR2+ hDCIS.01 xenografts. One reason for these differences may be because CCL2 levels were not yet saturated in the patient derived models, enabling CCR2 to respond to external CCL2 treatment. Since CCL2 was higher in CCR2+ h.DCIS.01 lesions, these lesions may have been less dependent on exogenous CCL2 than primary cells. Overall, these data demonstrated an important association between CCL2 levels and epithelial CCR2 expression to DCIS progression.

There were some phenotypic differences between the primary and hDCIS.01 MIND lesions. Primary DCIS lesions tended to be smaller than hDCIS.01 lesions. These differences may be because primary human breast epithelial cells required more nutrients than immortalized breast epithelial cells to grow. These nutrients may not have been provided by the murine microenvironment. Molecular differences in the murine and human breast microenvironment could have affected growth and invasion of human breast epithelial cells established within the breast duct. For example, estrogen levels are significantly lower in mice compared to humans^37^, and estrogen regulates expression of IL8 (CXCL8), a human specific cytokine with oncogenic effects in the breast^38,39^. Murine specific growth factors expressed by stromal cells such as EGF bind with lower affinity to human receptors^40,41^. Future studies could assess the role of CCL2/CCR2 signaling on DCIS progression in humanized mice^42^.

Several questions remain regarding CCL2/CCR2 signaling in DCIS progression. The molecular mechanisms through which CCL2/CCR2 signaling regulates DCIS progression remain poorly understood. Ongoing studies in the laboratory indicate that CCL2/CCR2 regulates DCIS progression by mediating changes in metabolism in breast epithelial cells. Animal studies are currently in progress to investigate these possibilities in part by examining the effects of targeting CCR2 on DCIS progression in multiple breast cancer models.

We acknowledge some limitations to these studies. For one, patient DCIS samples were not matched to IDC cases. Additionally, a small number of patient DCIS cases were examined in the MIND model. These aspects prevented us from drawing conclusions about the predictive nature of the proposed CCL2/CCR2 signaling protein signature to DCIS progression. Future studies would include following DCIS progression in the MIND model using a larger number of PDX samples. It would also be important to examine expression of CCL2/CCR2 chemokine signaling proteins in a retrospective cohort of DCIS with outcome data, and prospective cohort of patient DCIS with follow-up studies on outcome. This distinction in protein expression between could prove predictive in stratifying DCIS patients into low and high risk for progression to IDC. If the CCL2/CCR2 multi-protein signature were found to be predictive, DCIS patients absent for this protein signature would face a lower risk of progression and could potentially undergo fewer harsh treatments. Patients positive for this signature might face a higher risk of progression, and therefore would be treated using strategies more tailored to the patient.

CCR2 is currently a therapeutic target of interest, with promising results in clinical trials of CCR2 pharmacologic inhibitors in the treatment of pancreatic cancer and diabetes^43–46^. Currently, in selecting candidates for CCR2 targeting, tissues are screened for the presence of CCR2+ immune cells. Here, we show that epithelial overexpression of CCR2 is biologically significant and could be a criterion used to select candidates for CCR2 targeting. Targeting the CCL2/CCR2 pathway in combination with existing treatment strategies could be effective in preventing or treating IDC, thereby improving patient survival.

## Supplementary Information

Supplementary Information.
