# Supplementary material for: Expression of CCL2/CCR2 signaling proteins in breast carcinoma cells is associated with invasive progression
Source: Sci Rep. 2021 Apr 22;11:8708. doi: 10.1038/s41598-021-88229-0 (PMC8062684; doi:10.1038/s41598-021-88229-0)
Supplement: Supplementary file 1 — Supplementary Information. [file 41598_2021_88229_MOESM1_ESM.pdf]

Expression of CCL2/CCR2 signaling proteins in breast carcinoma cells is associated with invasive progression

Wei Bin Fang[1*], Diana Acevedo[1*], Curtis Smart[1], Brandon Zinda[1], Nadia Alissa[1], Kyle Warren[1], Garth Fraga[1], Li-Ching Huang[2], Yu Shyr[2], Wei Li[3], Lu Xie[3], Vincent Staggs[4], Yan Hong[1], Fariba Behbod[1], Nikki Cheng[1**]

*equal contributing author

**Corresponding author

Email: ncheng@kumc.edu

[1]University of Kansas Medical Center, Department of Pathology and Laboratory Medicine, Kansas City, KS 66160

[2]Vanderbilt University Medical Center, Center for Quantitative Sciences, USA Nashville, TN 37232, USA

[3]Shanghai Center for Bioinformation Technology, Shanghai Academy of Science and Technology, Shanghai, P.R.China, 201203

[4]Health Services and Outcomes Research, Children's Mercy Hospital and Clinics; University of Missouri Kansas City School of Medicine, US

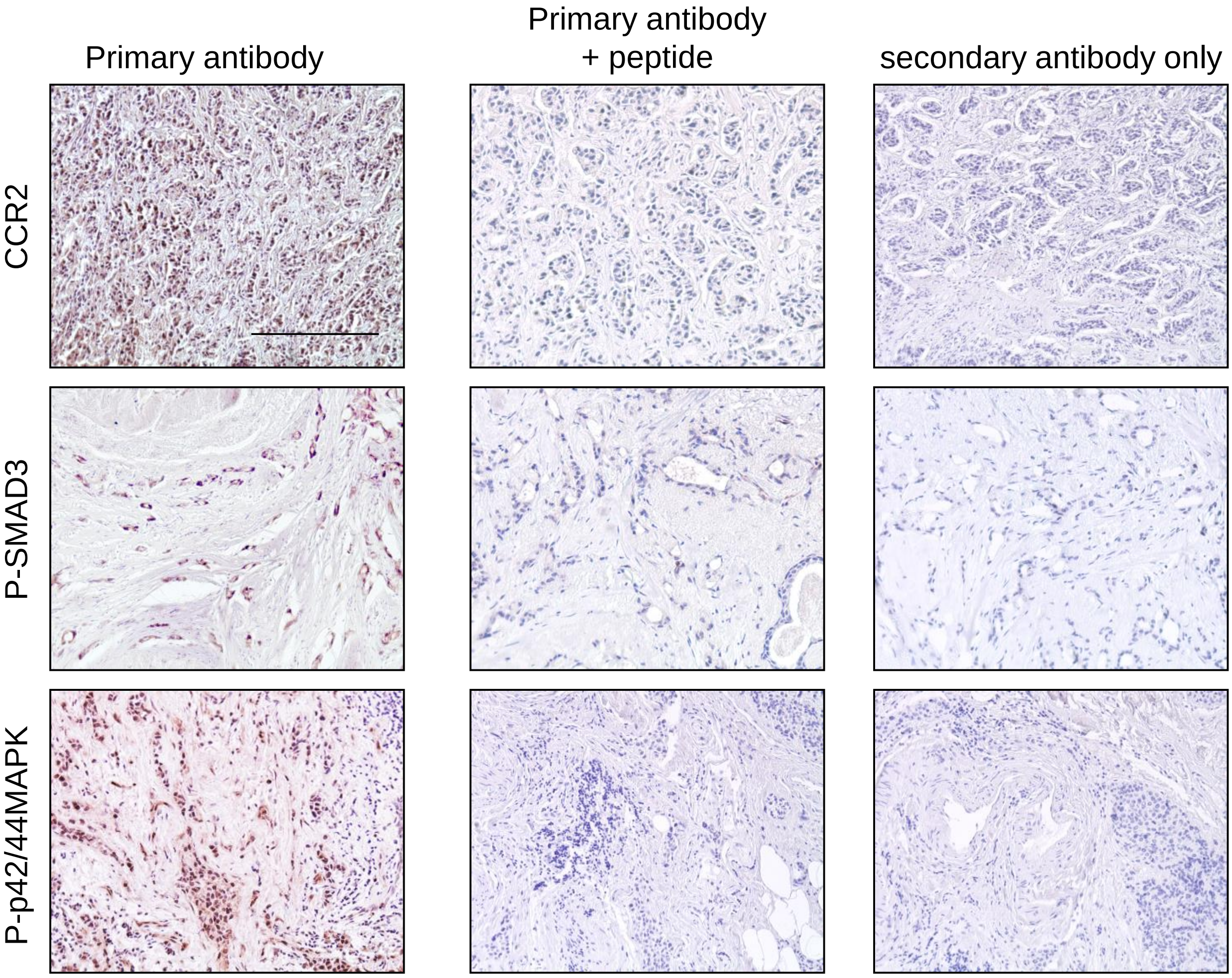

Supplementary Figure S1. Specificity of antibody staining. IDC tissue sections were immunostained with antibodies to indicated proteins, in the presence or absence of competitive peptide added at a 10-fold excess. Secondary anti-rabbit biotinylated antibody only were used for phospho-SMAD3, phospho-p42/44MAPK, and CCR2 immunostaining. Scale bar=100 microns.

A.

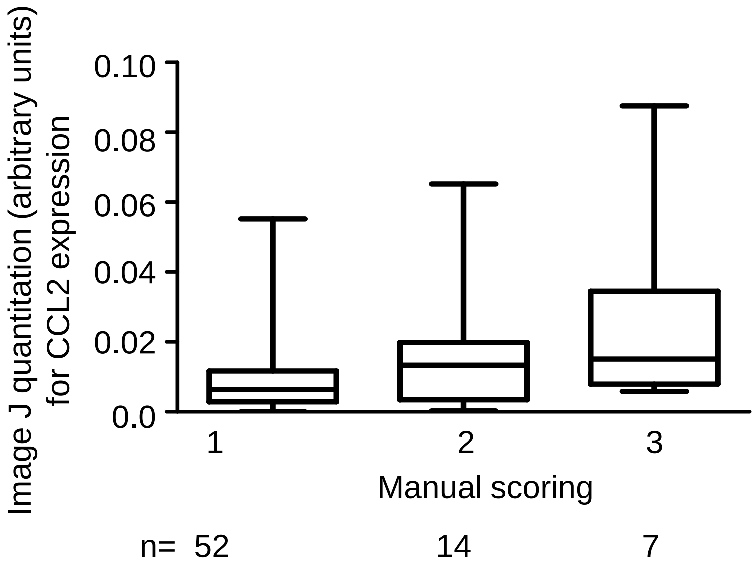

B.

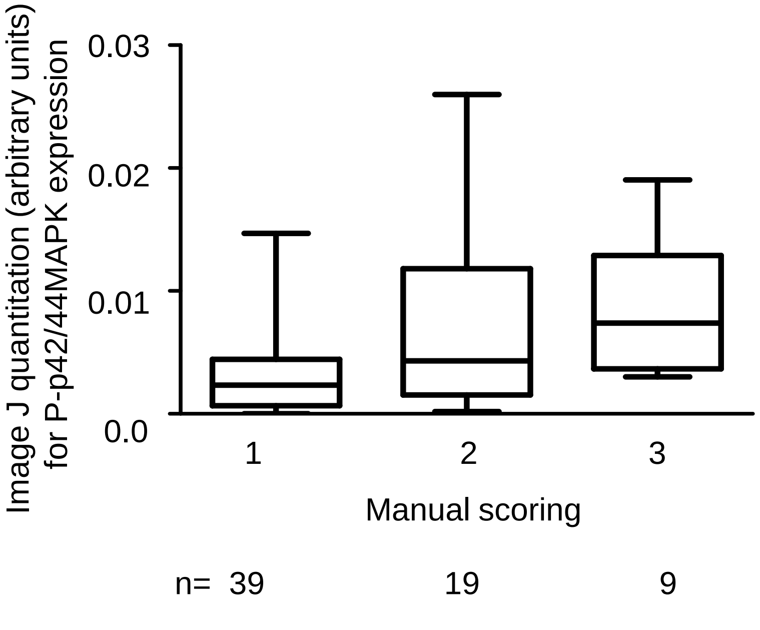

Supplementary Figure S2. Quantitation of immunostaining by Image J corresponds to manual scoring. Image J values for immunostaining of **A.** CCL2 or **B**. phospho-p42/44MAPK were cross referenced to manual scores of negative/weak (1), moderate (2) or strong (3) staining. Whisker box plots are shown indicating min and max values. Box indicates upper and lower quartile range. Line indicates median.

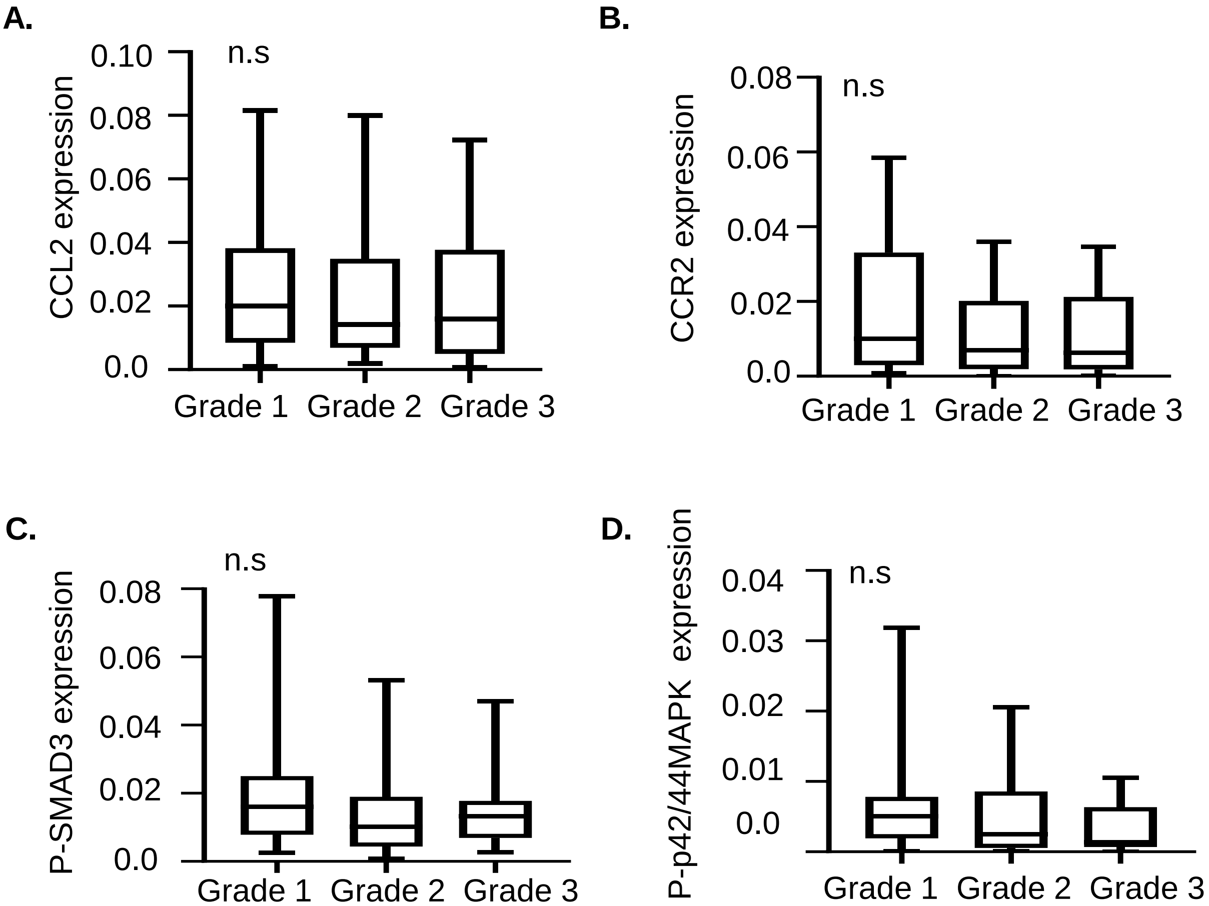

Supplementary Figure S3. CCL2/CCR2 signaling protein expression in IDC is not associated with histologic grade. Protein expression of **A.** CCL2, **B.** CCR2, **C**. phospho-SMAD3 and **D.** phospho-p42/44MAPK in IDC with grade 1 (n= 13), grade 2 (n=33), grade 3 (n=15) was quantified by Image J (arbitrary units). Whisker box plots are shown. Whiskers indicate min and max values. Box indicates upper and lower quartile range. Line indicates median. Statistical analysis was performed using Kruskal Wallis Test with Dunn’s post-hoc comparison.. Statistical significance was determined by $p<0.05$. n.s.= not significant.

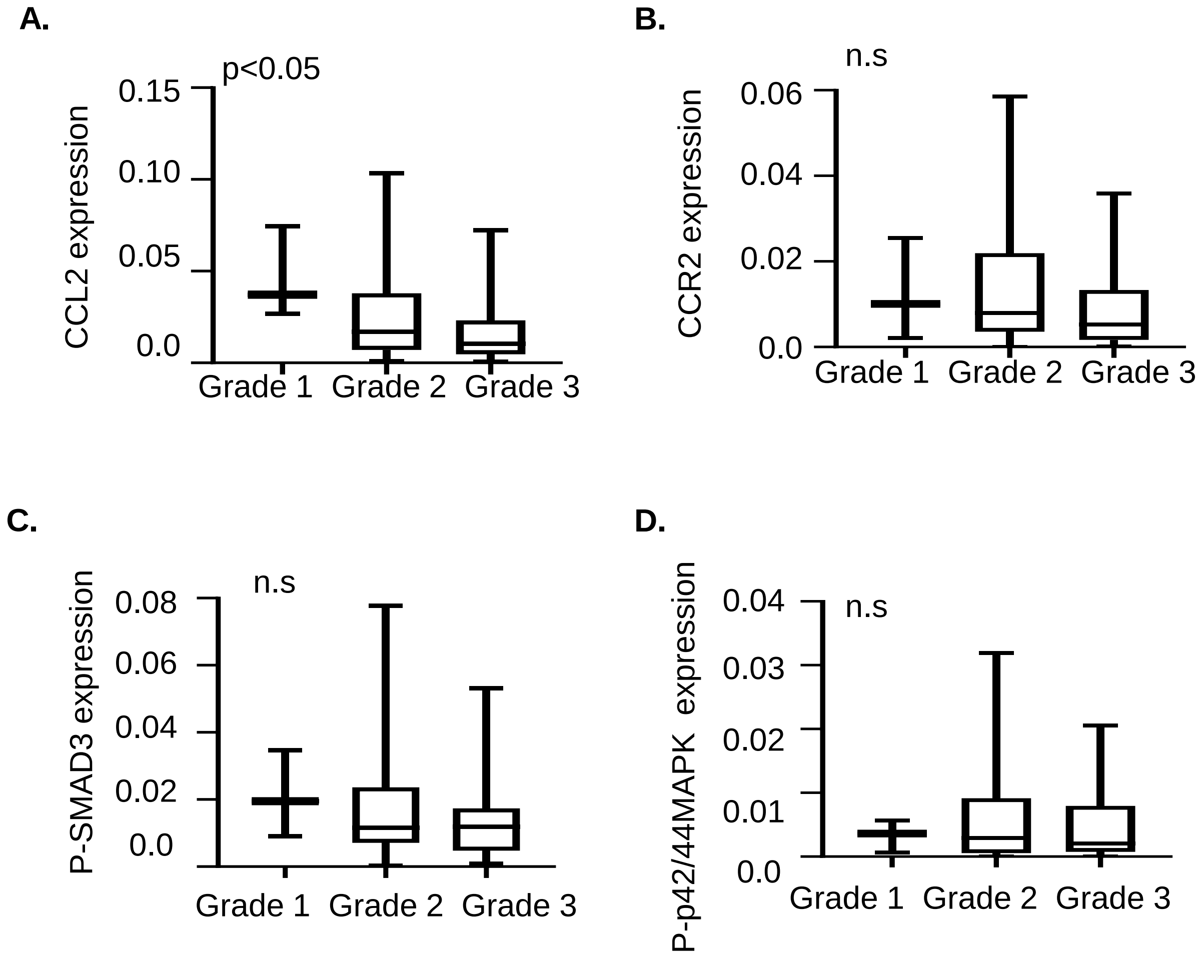

Supplementary Figure S4. There are few associations between CCL2/CCR2 signaling protein expression with nuclear grade in IDC. Protein expression of **A.** CCL2, **B.** CCR2, **C**. phospho-SMAD3 and **D.** phospho-MAPK in IDC tissues with nuclear grade 1 (n=3), grade 2 (n=39), grade 3 (n=32) was quantified by Image J (arbitrary units). Statistical analysis was performed using Kruskal Wallis Test with Dunn's post-hoc comparison. Whisker box plots are shown. Whiskers indicate min and max values. Box indicates upper and lower quartile range. Line indicates median. Statistical significance was determined by $p<0.05$. n.s=not significant.

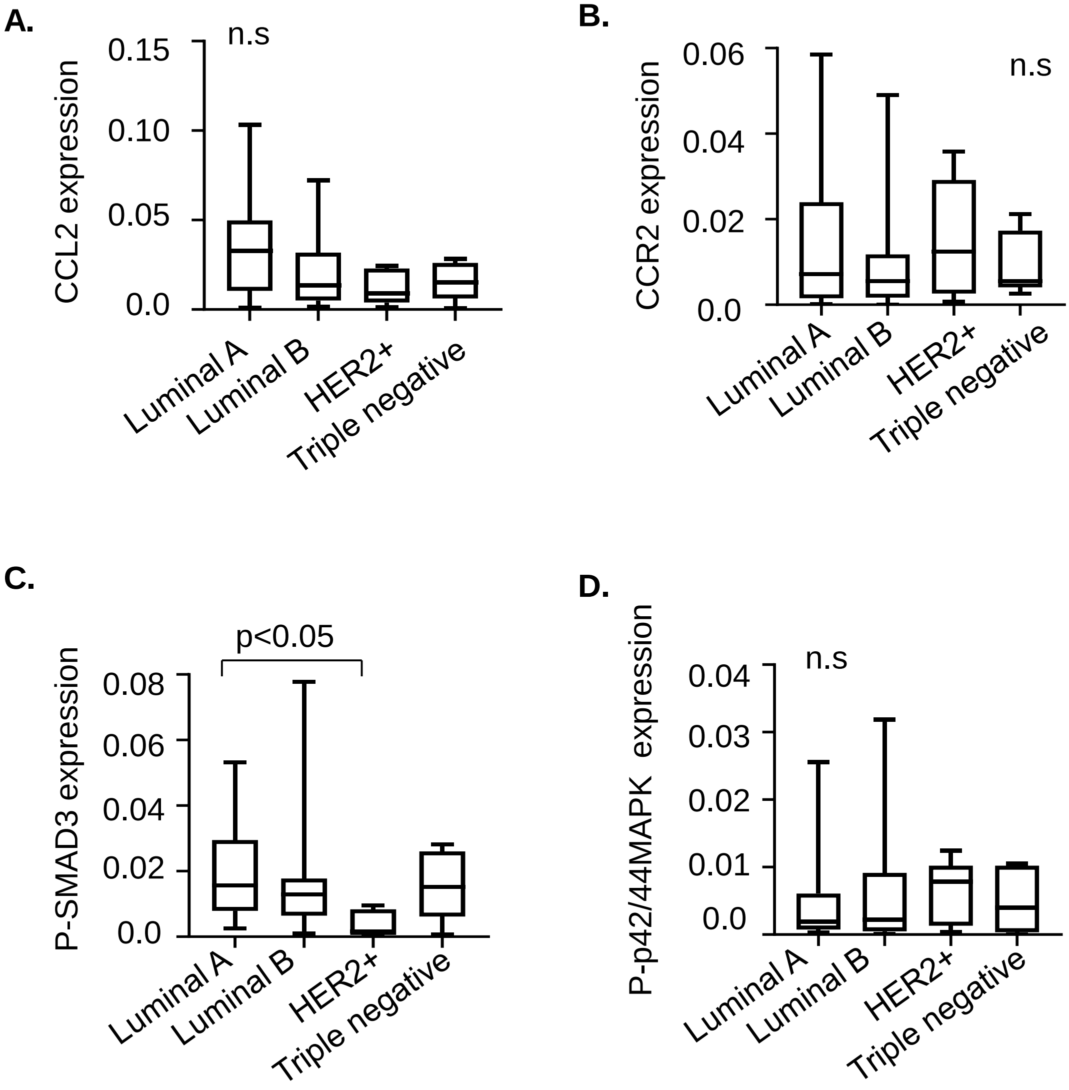

Supplementary Figure S5. CCL2/CCR2 signaling protein expression poorly associate with molecular subtype. IDC cases of luminal A (n=24), luminal B (n=39), HER2+ (n=5) and Triple negative (n=5) were assessed for protein expression of **A.** CCL2, **B.** CCR2, **C**. phospho-SMAD3 and **D.** phospho-p42/44MAPK. Expression was quantified by Image J (arbitrary units). Whisker box plots are shown. Whiskers indicate min and max values. Box indicates upper and lower quartile range. Line indicates median. Statistical analysis was performed using Kruskal Wallis Test with Dunn's post-hoc comparison. Statistical significance was determined by $p<0.05$. n.s=not significant.

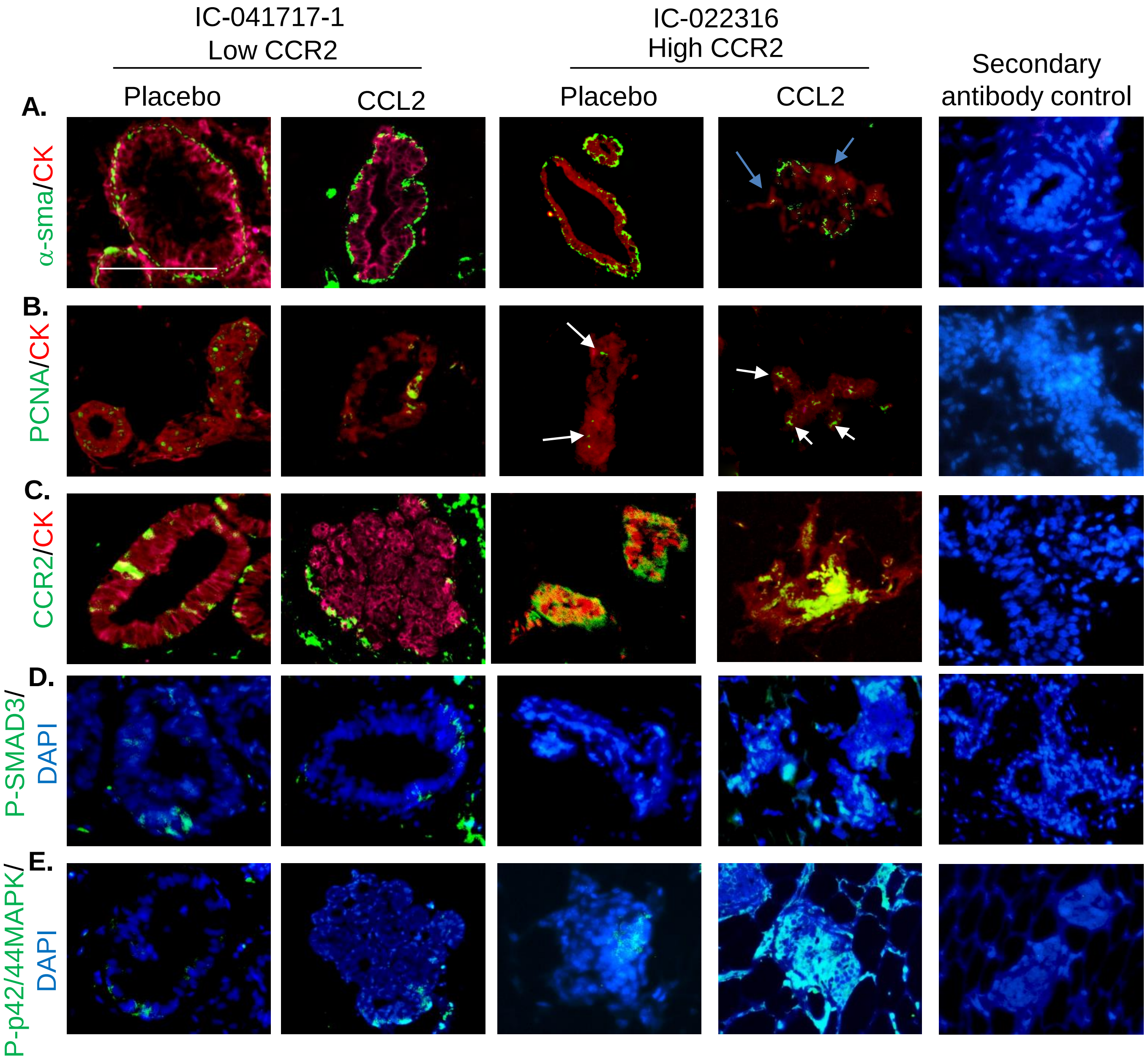

Supplementary Figure S6. Co-immunofluorescence staining of IC-041717-1 and IC-022316 primary MIND lesions. Primary MIND lesions were co-immunofluorescence stained for expression of CK5/19 (red) with: **A**. α-sma (green), **B**. PCNA (green), **C.** CCR2 (green), **D.** phospho-SMAD3 (green) or **E.** phospho-p42/44MAPK (green). Slides were counter-stained with DAPI. CK5/19 overlay is shown for α-sma, PCNA and CCR2 co-staining. DAPI overlay is shown for phospho-SMAD3 and phospho-p42/44MAPK staining. Blue arrows point to invasive cells. White arrows point to PCNA positive staining. Secondary antibody controls indicate secondary antibody rabbit and mouse antibodies over-layed with DAPI. Scale bar=200 microns.

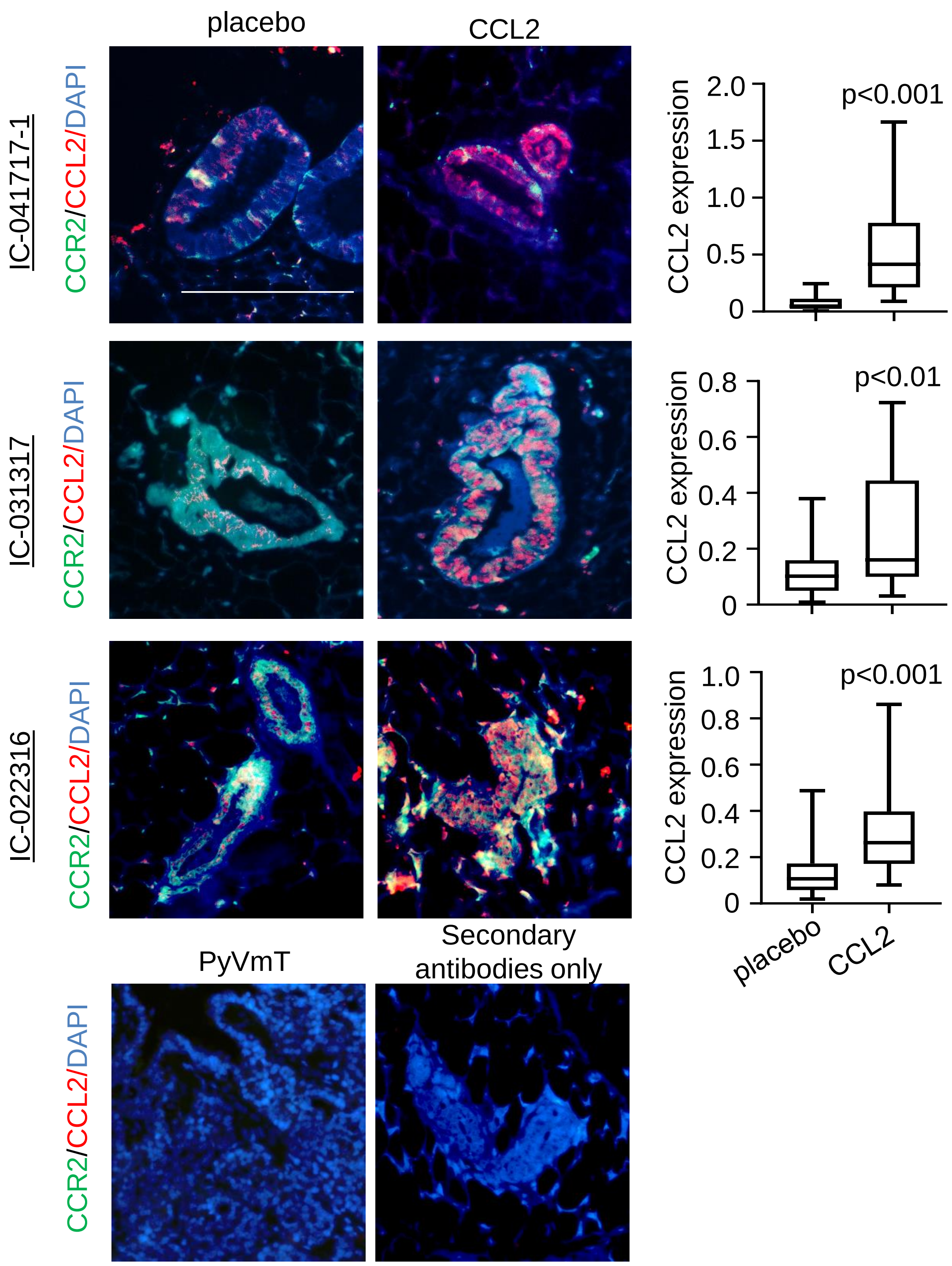

Supplementary Figure S7. Characterization of CCL2 levels in patient derived lesions established in the MIND model. Primary MIND lesions treated with or without CCL2 were co-immunofluorescence stained for expression of CCL2 (red) with CCR2 (green). PyVmT mammary tumor samples were used to control for species specificity of antibodies. Secondary antibody controls indicate secondary antibody rabbit and mouse antibodies over-layed with DAPI. Expression was quantified by Image J. Whisker box plots are shown. Whiskers indicate min and max values. Box indicates upper and lower quartile range. Line indicates median. Statistical analysis was performed using Two tailed T-test Statistical significance analysis was determined by $p<0.05$. Scale bar=200 microns.

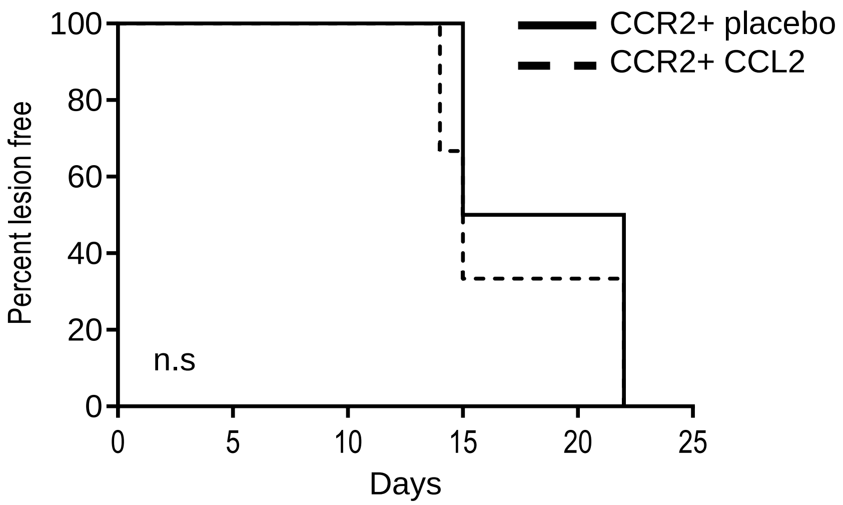

Supplementary Figure S8. CCL2 delivery does not affect development of mammary lesions from hDCIS.01 CCR2+ cells. hDCIS.01 cells sorted for CCR2 expression were injected in the mammary ducts of NOD SCID mice and treated with/without placebo or CCL2 protein. Mice were palpated for lesion formation. n=5 mice/group. Statistical analysis was performed using Log Rank (Mantel-Cox) test. Statistical significance was determined by $p<0.05$. n.s=not significant.

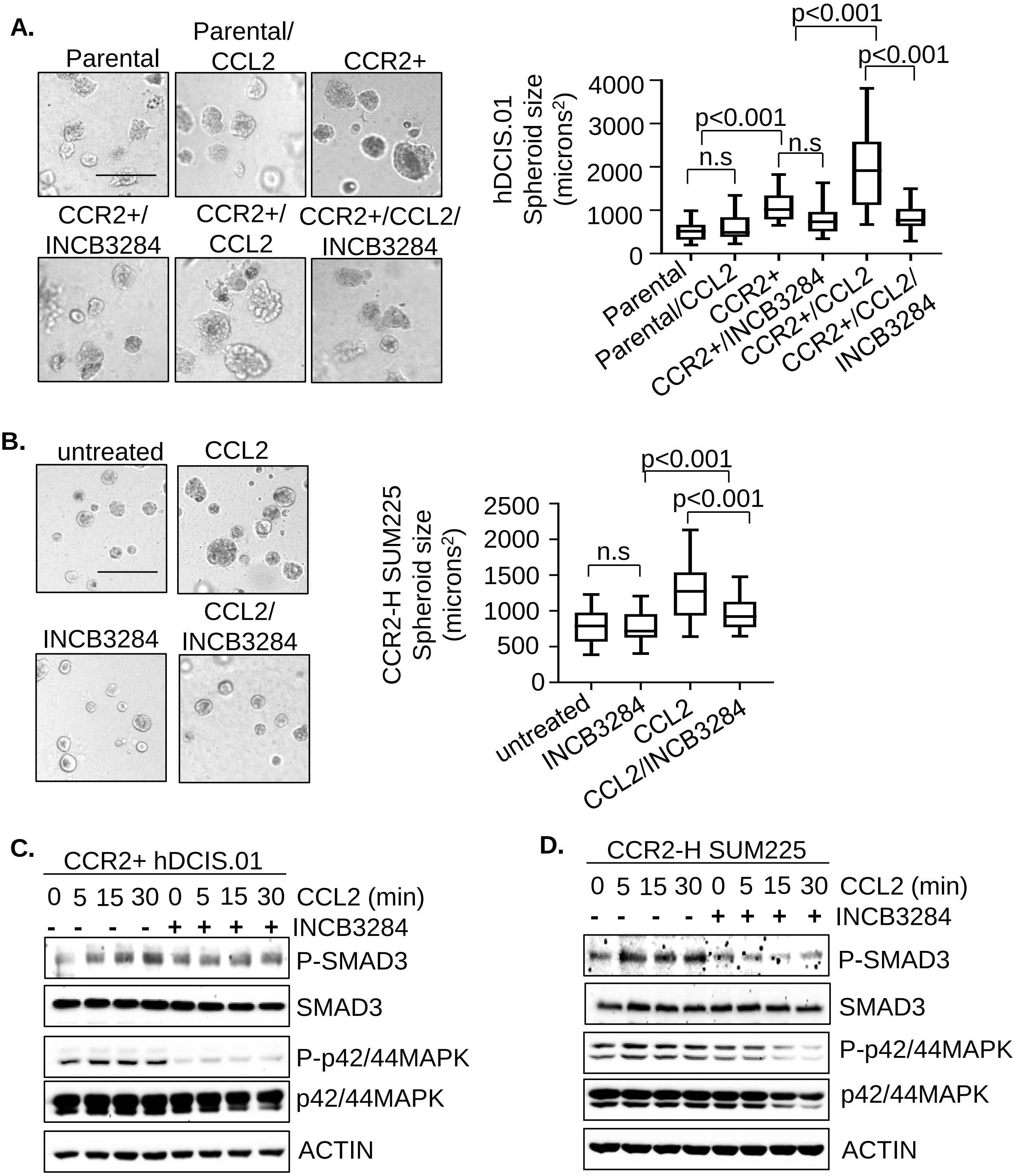

Supplementary Figure S9. Effect of INCB3284 treatment on hDCIS.01 and CCR2-H SUM225 cells. 3D cultures of **A.** Parental and CCR2+ hDCIS.01 cells or **B.** CCR2-H SUM225 cells were treated with/without 20 nM INCB3284 and/or 100 ng/ml CCL2 and analyzed for growth**.** Size of spheroids was quantified by Image J. **C.** CCR2+ hDCIS.01 cells or **D.** CCR2-H SUM225 cells were treated with/without 20 nM INCB3284 and/or 100 ng/ml CCL2 and analyzed for expression of the indicated proteins by immunoblot. Statistical analysis was performed using One Way ANOVA with Bonferroni post-hoc analysis. Statistical significance was determined by p<0.05, n.s=not significant. Scale bar=200 microns. full-length blots shown in Supplementary Figures S10-11.

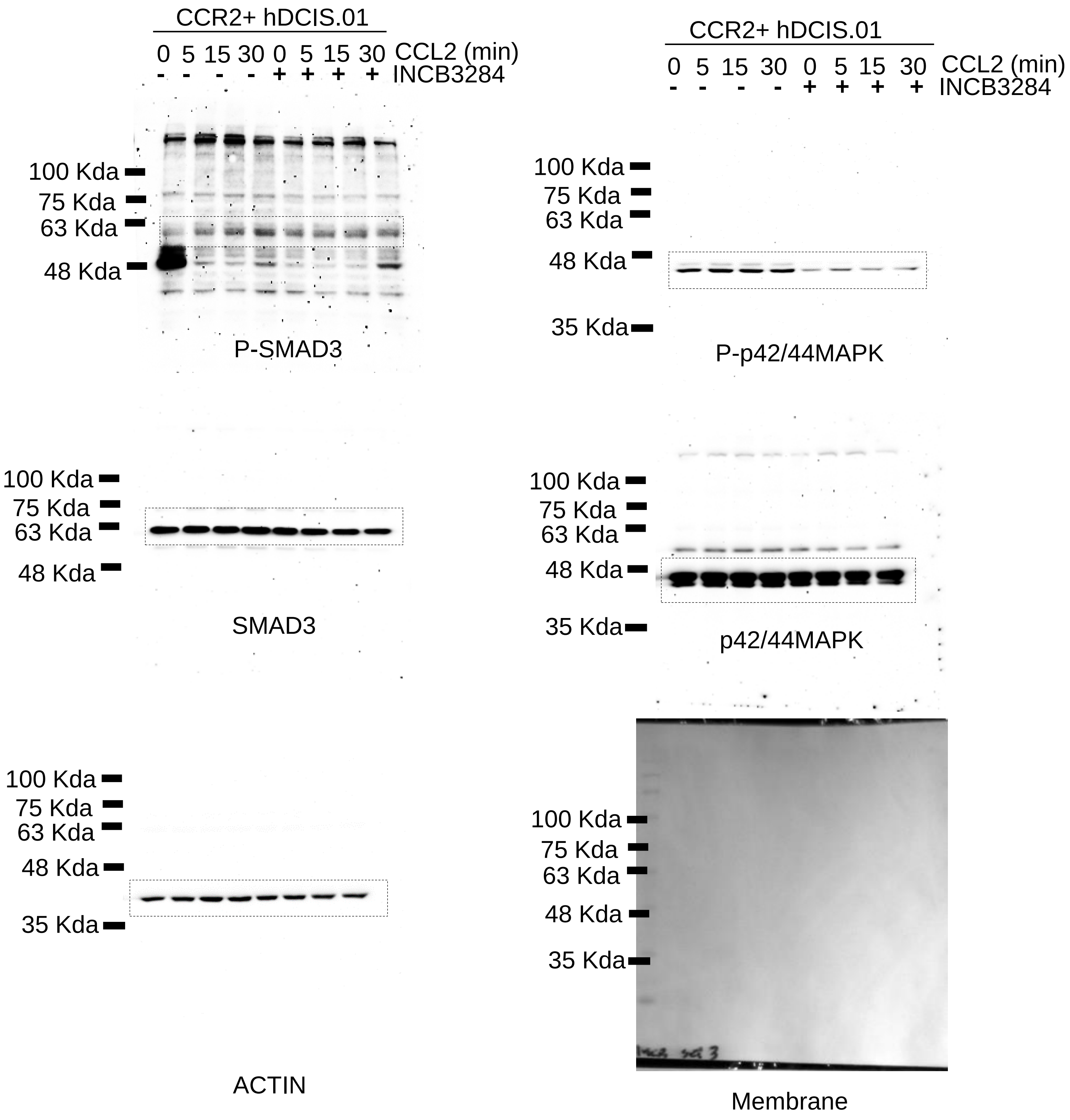

Supplementary Figure S10. Original full-length immunoblots of images presented in Supplementary Fig S9C. Images were captured using the UVP Imaging System. Brightness and contrast were adjusted for the entire blot using Adobe Photoshop. Cropped bands shown in 9C are outlined here.

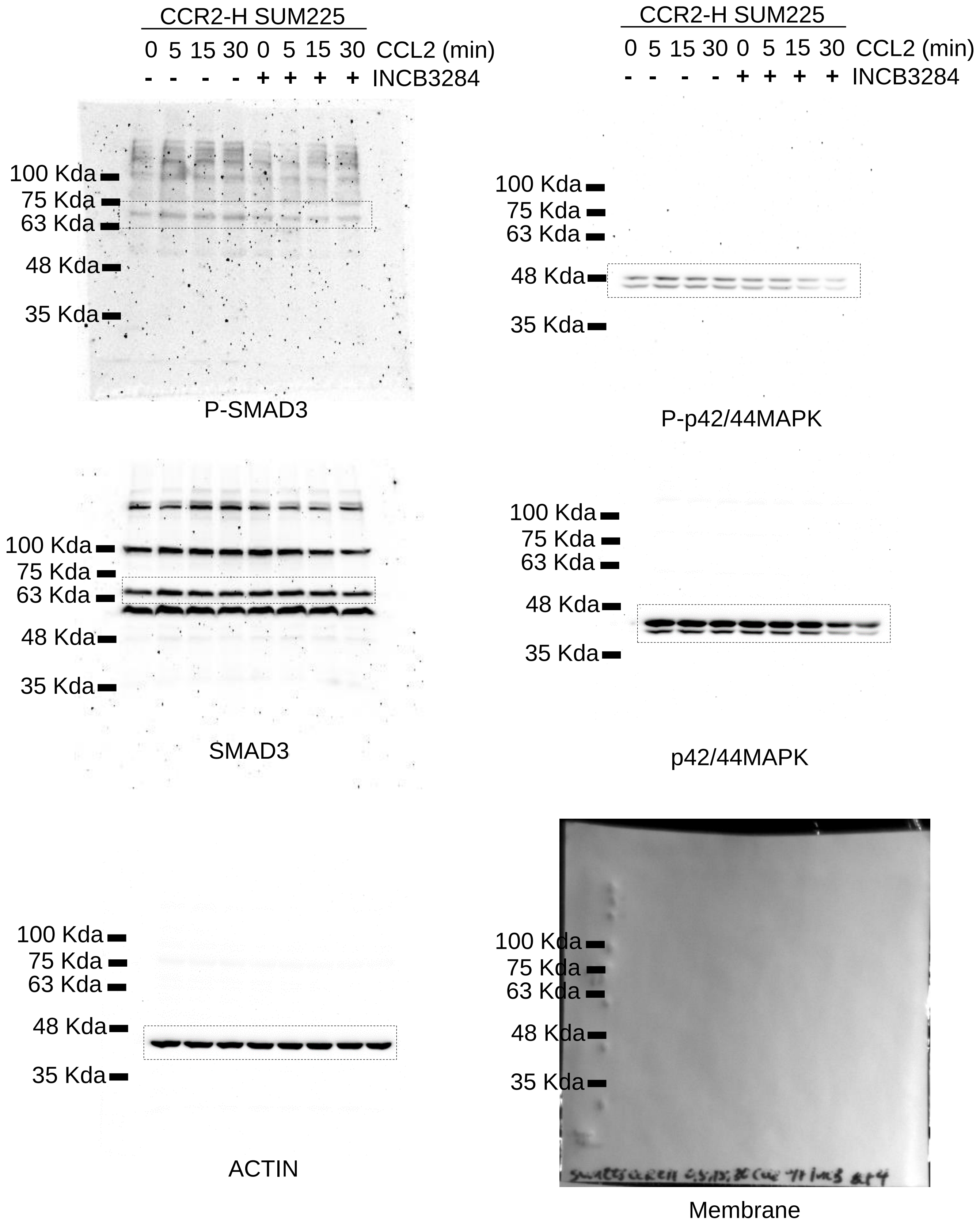

Supplementary Figure S11. Original full-length immunoblots of images presented in Supplementary Fig S9D. Images were captured using the UVP Imaging System. Brightness and contrast were adjusted for the entire blot using Adobe Photoshop. Cropped bands shown in 9D are outlined here.

Supplementary Table S1. KI67 and age are not associated with CCL2/CCR2 signaling protein expression in IDC patient samples. Associations were determined by Spearman correlation analysis. R= correlation coefficient. p= p-value. Statistical significance was determined by p<0.05. n.s =not significant.

	**CCL2**		**CCR2**		**P-SMAD3**		**P-p42/44MAPK**	
**Factor**	**R**	**p**	**R**	**p**	**R**	**p**	**R**	**p**
**Age**	-0.06	n.s	0.026	n.s	-0.21	n.s	0.12	n.s
**Ki67**	-0.04	n.s	-0.07	n.s	-0.02	n.s	-0.19	n.s
